# Human Induced Pluripotent Stem Cells: From Cell Origin, Genomic Stability, and Epigenetic Memory to Translational Medicine

**DOI:** 10.1093/stmcls/sxac020

**Published:** 2022-03-15

**Authors:** Mareike S Poetsch, Anna Strano, Kaomei Guan

**Affiliations:** Institute of Pharmacology and Toxicology, Technische Universität Dresden, Dresden, Germany

**Keywords:** human induced pluripotent stem cells, genetic stability, epigenetic memory, somatic origin, genetic aberrations, point mutations

## Abstract

The potential of human induced pluripotent stem cells (iPSCs) to self-renew indefinitely and to differentiate virtually into any cell type in unlimited quantities makes them attractive for in vitro disease modeling, drug screening, personalized medicine, and regenerative therapies. As the genome of iPSCs thoroughly reproduces that of the somatic cells from which they are derived, they may possess genetic abnormalities, which would seriously compromise their utility and safety. Genetic aberrations could be present in donor somatic cells and then transferred during iPSC generation, or they could occur as de novo mutations during reprogramming or prolonged cell culture. Therefore, to warrant the safety of human iPSCs for clinical applications, analysis of genetic integrity, particularly during iPSC generation and differentiation, should be carried out on a regular basis. On the other hand, reprogramming of somatic cells to iPSCs requires profound modifications in the epigenetic landscape. Changes in chromatin structure by DNA methylations and histone tail modifications aim to reset the gene expression pattern of somatic cells to facilitate and establish self-renewal and pluripotency. However, residual epigenetic memory influences the iPSC phenotype, which may affect their application in disease therapeutics. The present review discusses the somatic cell origin, genetic stability, and epigenetic memory of iPSCs and their impact on basic and translational research.

## Graphical Abstract

**Graphical Abstract:**
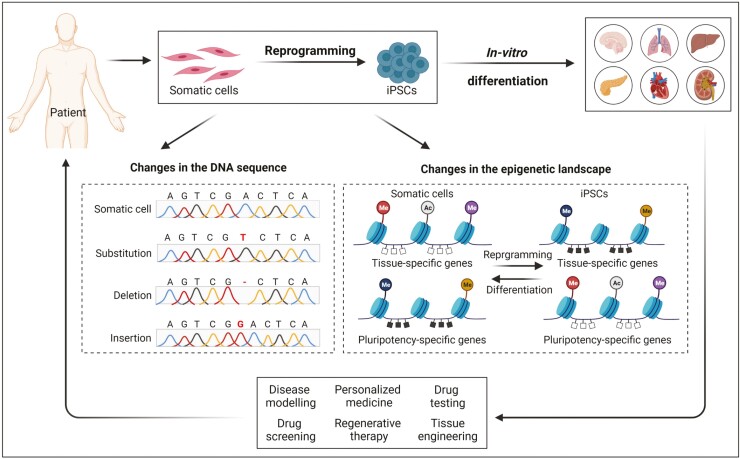

**Significance Statement**

Although the use of human iPSCs is rapidly expanding in basic and in translational and clinical research, questions remain about their safety due to potential mutation loads or epigenetic memory of the parental somatic cells. This review gives an outline of available cell sources routinely used for the generation of human iPSCs and discusses how the genomic stability and epigenetic memory are affected by somatic cell origins and by the reprogramming process. We anticipate that regular genetic screening of iPSCs should become a standard procedure to safeguard their use for clinical applications by sorting out iPSCs with high mutation loads or with incompletely erased epigenetic memory if unwanted.

## Introduction

In 2006, pluripotency in murine fibroblasts was successfully induced using the genome-integrating retroviral transduction of 4 transcription factors, Octamer 3/4 (Oct3/4), SRY-box containing gene 2 (Sox2), Krüppel-like factor 4 (Klf4), and the protooncogene cytoplasmic Myc protein (c-Myc) (collectively referred to as OSKM).^1^ The generation of human-induced pluripotent stem cells (iPSCs) from somatic cells offers great potential and the possibility to revolutionize the fields of stem cell biology, disease modeling, drug discovery, and regenerative medicine. Similar to human embryonic stem cells (ESCs), human iPSCs display all the important properties of unlimited self-renewal and pluripotency, including the capability to differentiate into numerous amounts of any differentiated cells in the human body.^2,3^ As human iPSCs are generated without the destruction of an embryo, existing ethical issues associated with human ESCs can be overcome and patient-tailored pluripotent stem cells with the potential for personalized cell replacement therapy can be obtained.

To make the best use of iPSCs, many efforts have been made over the past 15 years to compare iPSCs with ESCs and to shed light on the biological peculiarities of iPSCs. One of the important critical points is the choice of somatic cell source for reprogramming, which may influence the reprogramming efficiency and lead to the generated iPSCs holding epigenetic memory and mutations of their parental cells. In this review, we aim to discuss the somatic cell origin, genetic stability, and epigenetic memory of iPSCs, and provide an up-to-date overview of how these aspects influence the differentiation potential and applications of human iPSCs in translational medicine.

## Somatic Origin of Human iPSCs and Reprogramming Methods

**Figure 1.**
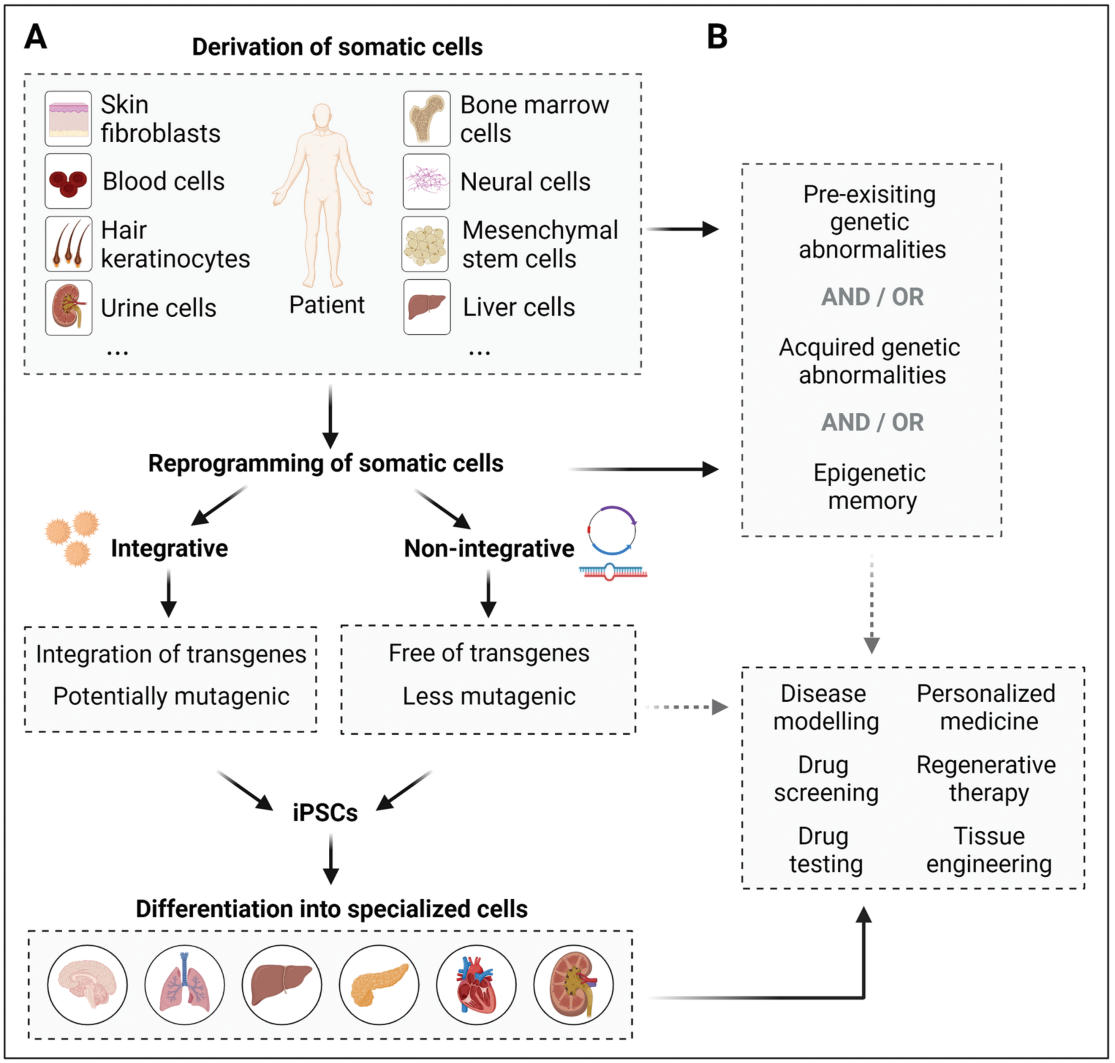
Process toward reprogramming of somatic cells to generate patient-specific iPSCs and application fields of iPSC-derived specialized cells. (**A**) Generation of iPSCs from a variety of somatic cell types by using integrative or non-integrative reprogramming approaches. Reprogramming of somatic cells by integrative strategies yields iPSCs with the integrations of transgenes into the genome, which may possess an increased mutagenic potential and are therefore considered unsafe. Alternatively, non-integrative strategies for reprogramming yield iPSCs, which are free of transgenes and are considered safe. Overall, the generated iPSCs can be differentiated into specialized cells and used as a tool for disease modeling, personalized medicine, regenerative therapy, and tissue engineering, in addition to their use for drug screening or drug testing. (**B**) Pre-existing genetic abnormalities of somatic cells can, when remaining undiscovered in the generated iPSCs, seriously limit their utility and safety for clinical or regenerative therapy. Genetic aberrations could be acquired during the process of reprogramming or due to extended passaging of iPSCs, which likewise limit their utility and safety. Therefore, to warrant safety of iPSCs for clinical applications, analysis of genetic integrity should be carried out on a regular basis. Furthermore, epigenetic memory of the somatic cells in iPSCs may influence lineage-specific differentiation and with-it utility and safety for clinical use.

Since the pioneering work by Takahashi and Yamanaka, somatic cells from a plethora of tissues have been used for the generation of human iPSCs with varied reprogramming efficiencies up to 4%,^4-6^ largely depending on the type and differentiation status of the cells and the method used for the reprogramming. Most available and commonly used somatic cells include skin fibroblasts, hair keratinocytes, mononuclear cells from peripheral or umbilical cord blood (including B and T lymphocytes, and CD34^+^ cells), and urine cells containing renal tubular epithelial cells and fibroblast-like or urothelial cells.^7^ Even cells harvested from biological waste materials were successfully used for reprogramming, including bone marrow cells, mesenchymal stem cells derived from fat tissue and teeth, liver and stomach cells, β-cells, melanocytes, or neural stem cells and progenitors^6^ (Fig. 1). This list of somatic cell sources is growing, including endothelial cells and cardiac progenitors from fetal tissues,^5^ human anterior cruciate ligament cells,^8^ myoblasts, ovarian follicular granulosa cells, amniotic fluid stem cells, and so on,^7^ indicating that cells of almost all tissues can be used for the generation of iPSCs (for details, see Refs. ^6,7^).

Although somatic cells with different tissue origins have been reprogrammed into iPSCs, the reprogramming process is highly inefficient with only a minority of donor cells being reprogrammed to pluripotency. Previous studies demonstrate that reprogramming is a continuous, but not well-understood, stochastic process and that the number of cell divisions is a key parameter driving epigenetic reprogramming to pluripotency.^9^ In addition, successful and efficient generation of iPSCs depends on the age of somatic cells used. A study by Lapasset et al revealed that reprogramming of senescent cells and cells from the elderly required a 6-transcription factor cocktail, containing LIN28 and NANOG in addition to OSKM.^10^

Currently, blood cells and skin fibroblasts are the most commonly used cell types for reprogramming because (1) they are easy to obtain, (2) conditions for the initial culture of these cells are well established, (3) reprogramming methods for these cells are successfully standardized, and (4) iPSC banking has been combined with blood or skin biopsy/fibroblast banking. However, since iPSCs retain the genetic information of the parental somatic cells, which may contain genomic aberrations (see part “Genetic stability”), and appear to have an epigenetic memory for the tissue of origin, which may influence lineage differentiation propensity of iPSCs (see part “Epigenetic memory”), cellular origin of iPSCs should be carefully considered before application in translational research.

Over the last 15 years, in addition to lenti- or retroviral-mediated, integrative transgene delivery strategies, many different methods of introducing exogenous reprogramming factors into the cell have been established to improve reprogramming efficiency and to generate transgene-free iPSCs for potential iPSC-based cell therapy (Fig. 1). Such methods include delivery of transgenes by using non-integrating viral approaches (eg, adenovirus and Sendai virus), or non-viral methods such as episomal vectors, mini-circle DNA vectors, piggyBac transposons, synthetic mRNAs, or recombinant cell-penetrating proteins.^11,12^ The Sendai viral (SeV) system provides long-lasting transgene expression after being introduced into target cells in a single delivery step, which allows many different cell types to be reprogrammed with considerable efficiency^11^ and is therefore advantageous for disease modeling. However, when applying iPSCs in the clinical field, it is necessary to screen for the presence of any trace of SeV backbone or transcript, which can be lasted for ten passages.^13^ The auto-erasable replication-defective and persistent Sendai virus system responding to mircroRNA-302 (SeVdp-302L) might overcome this problem,^14^ facilitating the generation of transgene-free iPSCs.

So far, episomal Orip/EBNA1 vectors have been used in iPSC-based clinical cell therapy trials.^15,16^ However, there are safety concerns due to the combined expression of EBNA1, L-MYC, and p53-repressing short hairpin RNA (shp53). In addition, episomal vectors persisted in iPSCs for at least 10 passages^17^ and bacterial-derived CpG motifs on the plasmid DNA could cause an immune response. These disadvantages make their clinical use challenging. To overcome these obstacles, doggybone DNA vectors containing the same expression cassettes without both OriP/EBNA1 and shp53 and lacking bacterial sequences were developed, which may reduce the immune response.^18^ Whereas reprogramming using cell-penetrating proteins suffered from extremely low iPSC induction efficiency and requirement of proteins in large quantities,^19,20^ use of synthetic modified mRNAs, which can be cost-effectively produced on a large scale, provides the clearest solution to generate the most unambiguously footprint-free iPSCs suitable for the clinic application.^21^ However, reprogramming using synthetic modified mRNAs requires serial transgene delivery, and blood cells seem to be difficult for reprogramming using the modified mRNA technology.^22^ The co-delivery of microRNA-302s/367 with synthetic modified mRNAs into fibroblasts promoted the generation of iPSCs with a high efficiency,^23^ which might be tested in blood cells.

Different from the conventional reprogramming approaches mentioned above, CRISPR-Cas9-based gene activation (CRISPRa) is a powerful tool for cellular reprogramming by inducing direct transcriptional activation of endogenous loci. Simultaneous targeting of endogenous pluripotency genes such as OSKM and LIN28A as well as genomic control elements like human embryonic genome activation (EGA)-enriched Alu-motif (EEA-motif) enables the efficient generation of iPSCs from human skin fibroblasts with reduced off-target gene activation and heterogeneity suitable for disease modeling and therapeutic application.^24^ This approach could be further improved by targeting the microRNA-302/367 locus, showing that both human lymphoblastoid cell lines and primary skin fibroblasts could be efficiently reprogrammed.^25^ All these recent advancements may make the use of iPSCs in personalized cell replacement therapy feasible and more reliable (Fig. 1).

## Genetic Stability

**Figure 2.**
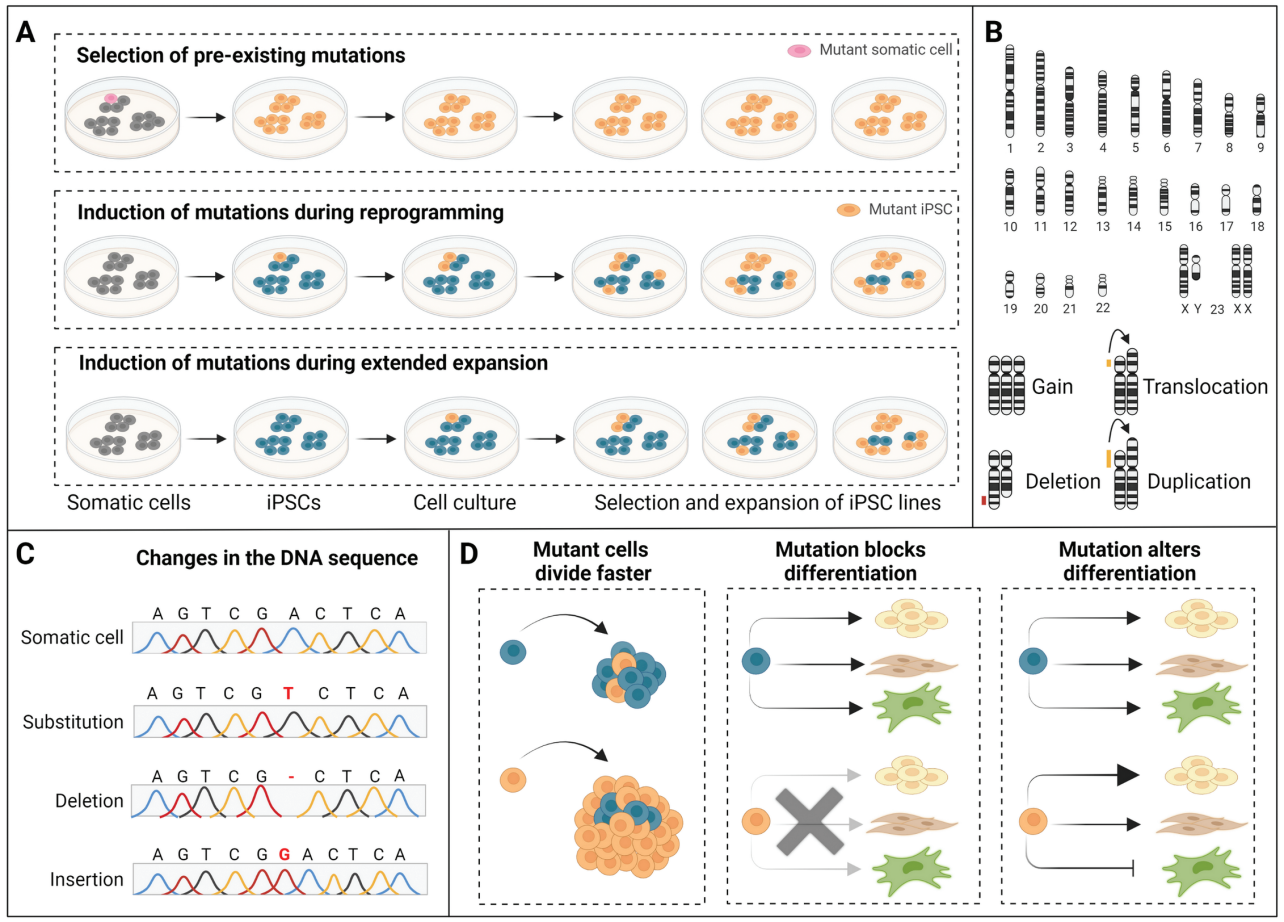
Sources and consequences of genomic instability in iPSCs. (**A**) Genetic alterations in iPSCs mainly arise via 3 routes: (i) Mutations are already present in the parental somatic cells from which iPSCs are derived and are subsequent cultured and expanded (upper panel), (ii) mutations can be induced during the process of reprogramming (middle panel), and (iii) mutations can be induced during extended passaging and prolonged culturing (lower panel). (**B**) Chromosomal rearrangements commonly observed in iPSCs, including gain of whole chromosomes, translocation of a chromosomal part from one to another chromosome, deletion of a chromosomal part, and duplications of a chromosomal part. (**C**) Changes in the DNA sequence commonly observed in iPSCs, including single nucleotide variation, ie, substitution of a single nucleotide at a specific position in the genome by another single nucleotide, and loss (deletion) or gain (insertion) of a single nucleotide. (**D**) Cell autonomous and cell interaction consequences of iPSC mutant variants, including growth advantage of the mutant variant as a result of faster cell cycle (left panel), block of differentiation by the mutant variant (middle panel), and alteration of differentiation patterns by the mutant variant (right panel).

For the iPSC application in disease modeling and regenerative medicine, it is pivotal for human iPSCs to possess genetic integrity and stability. However, the reprogramming process and the indefinite expansion of iPSCs afterward are linked to the acquisition of genomic aberrations, including chromosomal aneuploidy, sub-chromosomal copy number variants (CNVs), and point mutations. These may provide mutated iPSCs with a growth advantage in cell culture, influence their successful recapitulation of disease phenotypes and mechanisms, and limit their application in tissue regeneration (Fig. 2).

### Chromosomal Aneuploidy and Sub-chromosomal CNVs in iPSCs

Many studies reported that most human iPSC lines maintained normal karyotypes as their parental somatic cells as demonstrated using standard cytogenetic procedures (G-banding). However, some iPSC lines displayed karyotypic abnormalities. Instability of chromosome 12, manifesting as trisomy 12, accounts for approximately 46% and 30% of cases in aneuploidy of human ESCs and iPSCs, respectively. Since the chromosome 12 harbors cell cycle-related genes and the pluripotency-related gene *NANOG*, trisomy 12 confers a selective proliferation advantage in human pluripotent stem cells, in particular after long-term culture. Trisomy 17 and trisomy X were found more commonly in human ESCs than in human iPSCs.^26-29^ Other types of whole chromosomal aneuploidy in human iPSCs are trisomy 8 and trisomy 20q, which are similar to those of human ESCs and are regardless of the somatic origin and reprogramming procedure,^26,27^ but rather due to the in vitro long-term cultivation.

Sub-chromosomal CNVs are alterations in the copy number of a specific DNA region, which appear as amplifications or deletions. A recent meta-analysis of genetic abnormalities in human ESCs and iPSCs reported 738 recurrent genetic abnormalities in more than 100 different research articles from many different laboratories, including gains of chromosome and sub-chromosomal CNVs of various lengths.^30^ There were no recurrent genetic abnormalities detected in chromosome 2, 4, 10, or 21, according to the defined criteria that an abnormality shares part of its abnormal sequence with other abnormalities reported in at least 5 different publications. Notably, 90% of all recurrent genetic abnormalities were found in 20 common regions.^30^ Amplifications of a small region of 20q11.21 have been reported as the most recurrent region for CNVs in both ESCs and iPSCs.^29^ The 20q11.21 region includes the anti-apoptotic gene *BCL2L1*, the pluripotency-associated gene encoding inhibitor of DNA binding 1, dominant negative helix–loop–helix protein (*ID1*), and the gene coding for DNA methyltransferase 3B (*DNMT3B*), which contribute to the desensitization of human pluripotent stem cells to damage caused by erroneous mitosis during the long-term culture.^31^ Moreover, unique CNV signatures for human iPSCs have been reported, for example, recurrent CNVs at 1q31.3 and 17q21.1 were shared by >25% of human iPSCs whereas the loss of 8q24.3 was more common in iPSCs than in ESCs.^26,29^ Since these CNVs were not detected in the parental fibroblasts, they seem to have originated from genetic instability during the programming or in vitro culture of human iPSCs. Several studies reported that the number of CNVs was higher in human iPSCs than in the corresponding parental cells regardless of the reprogramming methods used.^32,33^ Although integration-free reprogramming methods are the favorite methods to generate iPSCs with a lower incidence of genetic variations,^34^ piggyBac transposon-based reprogramming of somatic cells led to the occurrence of some CNVs in iPSCs at lower passages, which tended to disappear gradually due to a negative selective pressure during cell passaging.^32^ However, some CNVs might result from passage-related positive selection pressure that provides the cells with a growth advantage in culture.^35^ Notably, some CNVs in human iPSCs were shared with the alterations in their parental somatic cells.^26,36^

Besides chromosomal aneuploidy and sub-chromosomal CNVs, point mutations occur in human iPSCs at low frequency. On average, an iPSC line harbors approximately 10 point mutations in the protein-coding regions^37-39^ and hundreds to thousands of mutations in the whole genome.^40-43^ Based on their origin, point mutations in iPSCs can be classified as (a) pre-existing mutations in the parental somatic cells and (b) induced mutations during reprogramming and/or during extended passaging (Fig. 2).

### Selection and Expansion of Pre-existing Mutations in the Parental Somatic Cell

Point mutations rarely occur in expressed genes, but they can pre-exist as genetic abnormalities in parental somatic cells and are then passively fixed during reprogramming.^34,40,44^ Gore and colleagues reported that about half of the mutations found in human iPSCs pre-existed at low levels in their parental fibroblasts whereas the rest occurred during reprogramming or extended culture of iPSCs.^44^ A recent study of somatic mutations across 36 non-cancerous tissues from more than 500 people reported that somatic mutation profiles were tissue-specific and associated with a variety of cellular functions.^45^ Ultra-deep sequencing analysis showed that mutations existed only at low frequencies in a minority of parental somatic cells but were detectable after cloning,^40,44,46^ suggesting that they have been randomly captured during the generation of iPSCs (Fig. 2A). Therefore, most human iPSC lines derived from the same parental source did not share the same mutations,^44,47^ highlighting the stochastic nature of iPSC generation as mentioned above. In addition, a positive correlation between age and mutation burden in most tissues was observed.^45^ Lo Sardo et al showed that the donor age of the somatic cells was associated with the probability of genetic alterations in iPSCs.^48^ Genomic studies further revealed that human iPSCs derived from skin fibroblasts or endothelial progenitors exhibited characteristic C-to-T and CC-to-TT transitions (in vivo acquired somatic mutations) commonly observed in melanoma^37,42^ and the C-to-A transversion found in iPSCs generated from cultured endothelial progenitors is a putative imprint of culture-induced/oxidative damage in vitro.^37^ Interestingly, lower mutation frequencies were observed in liver stem cells when compared to hepatocytes from the human liver.^49^ These findings suggest that the use of young somatic cells or adult stem cells may lead to lower mutation loads in human iPSCs.

### Generation of Mutations During Reprogramming and Extended Culture

Several reports have shown that de novo point mutations can be introduced during reprogramming and extended culture independent of the reprogramming methods (integrative or non-integrative) and the parental cell source used.^44,47^ As the reprogramming process of somatic cells occasionally causes DNA double-strand breaks (DSBs), which can be induced by oxidative stress and replication stress, DNA repair mechanisms are involved in counteracting mutations occurring during reprogramming, such as error-free homologous recombination (HR) and non-homologous end-joining (NHEJ).^50,51^ A study showed that DSBs primarily occur in iPSCs at low passages due to extended reprogramming stress, while fewer DSBs were detected in iPSCs at higher passages.^52^ Comprehensive bioanalytical analysis of the mutagenic signatures in iPSCs by whole-genome sequencing and next generation sequencing revealed that accumulation of reprogramming-associated mutations, especially base substitutions, were caused by oxidative stress and subsequent DNA damage owing to the overexpression of reprogramming factors and a preferential use of error-prone repair mechanism.^37,43^ Strategies to specifically target p53 have been shown to improve efficiency to generate iPSCs, but at the cost of genetic instability.^53^ In line with this, a study by Laurent et al showed that deletions of tumor-suppressor genes could occur during reprogramming.^33^ Moreover, DNA interstrand cross-links caused by DNA-damaging endogenous metabolites, including reactive oxygen species and aldehydes, and followed by the formation of DSBs, are preferentially repaired by the Fancomi anemia pathway using HR to prevent genomic alterations during reprogramming.^54^ However, if remained unrepaired, such DNA lesions coincide with irreversible genomic changes and induction of apoptosis.

Genetic instability of iPSCs and activation of apoptosis resulted from replication stress are linked with the failure of checkpoint kinase 1 (CHK1) activation,^55^ which could be significantly reduced by supplementing nucleosides or antioxidants.^56,57^ Increasing the CHK1 levels could reduce reprogramming-induced replication stress and increase the efficiency of iPSC generation.^56^ Moreover, replacing c-MYC by cyclin D1 but maintaining OCT3/4, SOX2, KLF4 and LIN28 for reprogramming improved genomic stability of iPSCs by reducing cell stress and promoting DNA damage response by error-free HR.^58^

It is worth noting that a large fraction of de novo point mutations was located to transcriptionally repressed and structurally condensed lamina-associated heterochromatin domains, indicating that chromatin organization biased regional mutation rates in iPSCs.^59^ Most protein-coding point mutations were nonsense or non-synonymous mutations, or splice variants, which are particularly enriched in mutated genes or in oncogenic genes.^44^ The coding point mutations associated with reprogramming were maintained during extended culture, unlike those reported for some CNVs.

Although iPSCs in long-term culture preferentially use HR to cope with DNA damage,^50^ amplifications of tumor-promoting genes tended to occur during prolonged culture.^10^ Genetic aberrations in iPSCs could, at least partially, result from a transient G1/S cell cycle checkpoint deficiency,^55,60^ or lack of p53-mediated cell cycle arrest.^61^ Whole-exome sequencing revealed that the *TP53* mutant allelic fraction increased with passage number of human ESCs under standard culture conditions. All *TP53* mutations in human ESCs and iPSCs identified caused coding changes in the DNA-binding domain of p53.^35^ These findings suggest that *TP53* mutations confer selective advantage during long-term culture of human ESCs and iPSCs.

Taken together, pre-existing and de novo mutations that occur during or after reprogramming contribute to a high mutational load in iPSCs. Their expansion, which may go unnoticed, could greatly compromise the genetic stability of human iPSCs, and affect the differentiation efficiency, and thus the understanding of the disease-underlying mechanisms (Fig. 2). As unwanted mutations, particularly those arising during cell proliferation, might result in oncogenic transformation, human iPSCs that are intended for clinical use should therefore be free of cancer-associated genetic alterations.^43^ Although it remains elusive whether genetic aberrations represent an actual risk factor for adverse therapy, thorough characterization of iPSCs, frequent genomic monitoring, and optimization of derivation and culture conditions could promote genetic stability and safety of human iPSCs for clinical use.^42,62^

## Epigenetic Memory

Epigenetic mechanisms define cell type identity and function via conferring changes in the gene expression program without modifying the DNA sequence. Cellular reprogramming is a gradual process of epigenetic changes that include DNA methylation, histone tail modifications (acetylation and methylation), and incorporation of histone variants into chromatin, which can alter gene expression and cell properties and behavior (for more details, please also see reviews^11,12,63,64^).

**Figure 3.**
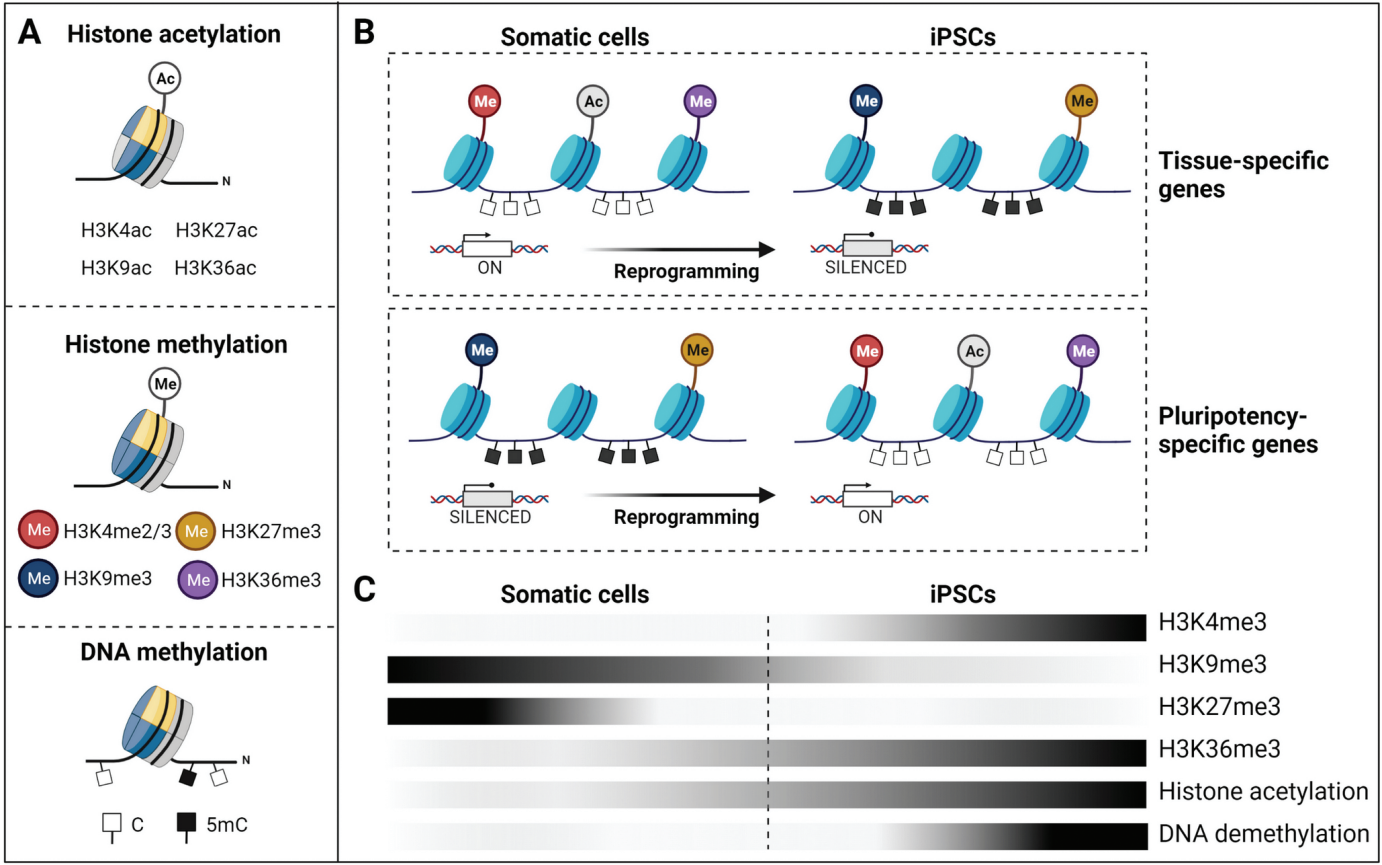
Changes of the epigenetic landscape occurring during generation of iPSCs. (**A**) Profound and intense modification of the histone tail, ie, histone H3 acetylation at lysine residues (upper panel) and histone H3 methylation at lysine residues (middle panel), in addition to DNA modification, ie, DNA methylation (black boxes) or DNA hypomethylation (white boxes) (lower panel). (**B**) Changes in the epigenetic landscape occurring during reprogramming of parental somatic cells with respect to tissue-specific (upper panel) and pluripotency-specific genes (lower panel). In somatic cells, tissue-specific gene promoters are demethylated and enriched for the active histone tail modification H3K4me3, by which they remain in an active state. Opposite, pluripotency-specific genes remain silenced by both DNA methylation and repressive H3K9me3 and H3K27me3. During reprogramming, silencing of somatic genes is directed by repressive H3K9me3, while activation of pluripotency-specific genes is instructed by H3K4me3 and H3K36me3, in addition to histone acetylation and promoter hypomethylation. (**C**) Epigenetic dynamics toward activation of pluripotency-specific genes occurring during successful reprogramming of somatic cells to iPSCs as indicated by a color gradient. Black colors of the bars indicate high abundance, white colors low abundance of prominent histone tail modifications or DNA demethylation patterns of pluripotency-specific genes.

DNA methylation involves the addition of a methyl group to a 5ʹ cytosine at cytosine-guanine dinucleotide (CpG) sites by DNA methyltransferases (DNMTs) to either activate or repress gene transcription. While DNMT3A and DNMT3B typically regulate de novo methylation of CpG sites, their absence does not affect human iPSC generation, suggesting that de novo DNA methylation is dispensable for cellular reprogramming.^65^ Maintenance of methylation patterns in the cell is regulated by DNMT1. Histone tail modifications, especially histone methylation, involve the addition of up to 3 methyl groups mainly to lysine residues of histone H3 protein. While transcriptionally silent genes are marked by H3K9 di- or trimethylation (H3K9me2/H3K9me3), di- or trimethylation at H3K4 (H3K4me2/H3K4me3) is associated with the activation of nearby genes.^66^ This delicate balance in chromatin modifications is sensitive to disturbances, which can result in reduced self-renewal, enhanced differentiation, and/or impeded reprogramming of somatic cells^63,64^ (Fig. 3).

Somatic cells possess a steady chromatin landscape that is characterized by highly condensed heterochromatin and repressive histone tail modifications.^66^ In contrast, pluripotent stem cells possess a unique epigenetic profile enriched for decompacted, euchromatic chromatin regions associated with activating histone tail modifications.^67^ During reprogramming, the donor cell-specific epigenetic landscape needs to be modified to allow activation of pluripotency-associated genes buried in densely compacted regions of heterochromatin. These events include (a) silencing of somatic cell-specific genes and (b) activation of pluripotency-associated genes and genes specific for cell cycle regulation^63,64,68^ (Fig. 3).

Pluripotency genes remain silenced in somatic cells by DNA methylation and repressive histone modifications, such as H3K27 trimethylation (H3K27me3), whereas in pluripotent cells the promoters of pluripotency genes are highly demethylated and show typical activating histone mark H3K4me3. During reprogramming, silencing of somatic genes is directed by permissive H3K4me2 and repressive H3K9me3. Activation of pluripotency genes, in turn, is instructed by trimethylation of both H3K4 (H3K4me3) and H3K36 (H3K36me3), in addition to histone acetylation and promoter hypomethylation.^69,70^ Mega base-scale chromatin domains enriched for H3K9me3 were identified in human fibroblasts, which blocked initial access of OSKM to the genome, and impeded the efficiency of reprogramming as being primarily located to genes required for pluripotency.^71^

It is worth noting that local and 3D chromatin architecture reflecting the position and density of nucleosomes as well as the presence of histone variants provide additional levels of gene regulation in iPSCs. Histone variants generally affect the ability of nucleosomes to undergo remodeling and to accommodate active or repressive histone modifications.^72^ The presence of the histone variant macroH2A in chromatin is associated with resistance to efficient chromatin remodeling in somatic cells. MacroH2A and especially the isoform macroH2A1 preferentially occupy genes that are expressed at low levels and are marked with H3K27me3, including pluripotency genes and bivalent developmental regulators. In this way, they act as an important epigenetic barrier to prevent the gain of H3K4me2 and protect somatic cells against reprogramming by maintaining pluripotency loci in a repressed state.^73,74^ For further details, please see a recent review summarizing the evidence for an important role of macroH2A in iPSC reprogramming.^11^ In contrast, histone variants H2A.X and H3.3 have been shown to facilitate reprogramming following nuclear transfer^75,76^ and H3.3 incorporation is required to fully maintain the pluripotent nature of ESCs.^77^ Given the prominent role of active and repressive histone variants during reprogramming, a better understanding of their function and deposition^78^ is likely to aid in the therapeutic application of reprogramming.

Characteristic histone modification and DNA methylation landscapes are correctly reprogrammed in most authentic human iPSC lines. However, they can be incompletely reset in partially reprogrammed iPSCs.^79,80^ In particular, although human iPSCs share the criteria for pluripotency with ESCs, many differentially methylated regions (DMRs), which are located primarily at CpG sites, have been identified in human iPSCs when compared to ESCs.^69^ Some DMRs are the result of incomplete reprogramming of parental cells, leading to somatic epigenetic memory. For example, human iPSCs derived from myeloid cells, hematopoietic cells, or insulin-producing β-cells retain at least in part their epigenetic memory,^20,81^ which result in biased differentiation. Kim et al showed that colony formation was higher in blood-derived iPSCs compared to iPSCs derived from non-hematopoietic cells. The former retained residual methylation at loci required for the hematopoietic fate,^20^ indicating that residual DNA methylation signatures influence the cell fate commitment. Studies on human iPSCs from 3 types of somatic cells (endothelial cells, fibroblasts, and cardiac progenitors) of the same individuals revealed that endothelial cell-derived iPSCs at early passages differentiated into endothelial cells with a higher efficiency than the other 2 types of iPSCs.^5^ Notably, the effects of epigenetic memory on the differentiation potential of iPSCs tend to disappear through extended passaging in long-term culture.^5,82^ The disappearance of differences among the different human iPSC lines correlated with the emergence of bivalent domains (ie, gene regions enriched in markers of both active and inactive chromatin) in differentiation-related genes marked by H3K4me3/H3K27me3.^12,82^ However, many DMRs were acquired de novo in human iPSCs during reprogramming.^20,69,81,83^ The presence of human iPSC-specific DMRs points toward the existence of genomic regions more prone to atypical methylation, which might influence the lineage differentiation potential and the application of iPSC derivatives in drug screening and disease modeling.^81^

Nevertheless, epigenetic memory retained from the tissue of origin has been used as an advantageous propriety in some studies. A recent study reported that human iPSCs maintained a residual epigenome of whole ganglionic eminence from which they were derived. This epigenetic memory allows producing striatal medium spiny neurons that share fundamental characteristics with whole ganglionic eminence to precisely enhance striatum fate differentiation and could therefore represent a useful alternative cell source for cell replacement therapy for Huntington disease.^84^ Another study using 5 cell types in the retina at 2 stages of development showed that the cells that were most difficult to reprogram made the best retina, reflecting their epigenetic memory.^85^ Furthermore, keratinocyte-derived iPSCs were more prone to form neuroectodermal structures compared to fibroblast-derived iPSCs.^86^ Fetal neural stem cell-derived iPSCs yielded higher number of neural precursors and more differentiated neuronal cells compared to fibroblast-derived iPSCs.^87^

Taken together, successful reprogramming of somatic cells into iPSCs largely depends on faithful reprogramming of the cell’s epigenetic landscape to implicitly shut down somatic gene expression in order to activate the pluripotency-related transcriptional program. This could be followed by the formation of DMRs, whose quantities may vary depending on the donor cells, methods used for reprogramming, as well as culture conditions. The majority of DMRs result from de novo aberrant methylation, with only a minority to arise as a consequence of epigenetic memory. Moreover, incomplete reprogramming can severely compromise the epigenetic status that could result in both variable gene expression and biological function among diverse iPSC lines. The donor cell-specific epigenetic memory of iPSCs can have a substantial impact on the directed lineage differentiation potential for applications in disease modeling, drug screening, or cell replacement therapy.

## Problems and Perspectives

Although human iPSCs become a routinely used in vitro system for disease modeling, drug screening, and personalized medicine, scientists unknowingly use iPSCs derived from a broad spectrum of somatic cells, which potentially harbor cancer-related mutations or a limited lineage-specific differentiation potential due to partly erased epigenetic memory. The fact that tumorigenic mutations or partially retained epigenetic gene activity among various iPSC lines have been observed in numerous studies suggests that reprogramming and culture conditions should be more precisely explored to reduce the selection pressure. Importantly, the discovery of undisclosed mutations has already forced the halt of clinical trials.^88^ Therefore, genome-wide analyses of human iPSCs on a regular basis could help to detect potentially harmful mutations at very early stages and would easily guide exclusion of these cells from therapeutic applications. Genome-wide analyses should be tackled at key main steps, ie, during the initial selection of somatic cells used for reprogramming, during the iPSC characterization, long-term culture and subsequent lineage-specific differentiation, and in the late stages to ensure the safety of the transplantation of iPSC-derived cellular products.^35^ In addition, more studies to include an even larger number of human iPSC lines are needed to search for genetic variations and to discriminate those that are harmless from the ones that pose clinical risks.

## Conclusion

Induced pluripotency represents a breakthrough in biomedical science, offering nowadays a powerful tool for disease modeling, drug screening, personalized medicine, and tissue or organ-specific regeneration. Although human iPSCs possess—at least to some extent—various epigenetic and transcriptional differences compared to human ESCs, it is still under debate whether these dissimilarities to ESCs functionally impact their differentiation potential. To warrant the utility and safety of human iPSCs for disease modeling and clinical applications, regular screening of genetic integrity should become a standard procedure to screen out iPSC lines with high mutation loads or those harboring potentially deleterious mutations in genes essential for development. Moreover, epigenetic memory retained from the tissue of origin may be used as an advantageous propriety of human iPSCs to produce the best cells for transplantation.

## Data Availability

No new data were generated or analyzed in support of this research.
